# The effect of cuff arthropathy stage on sleep disturbance and kinesiophobia in reverse shoulder arthroplasty patients

**DOI:** 10.1186/s12891-024-07338-9

**Published:** 2024-03-23

**Authors:** Gokhan Ilyas, Ercument Egeli, Fikri Burak Ipci, Oguzhan Gokalp

**Affiliations:** 1 Faculty of Medicine, Department of Orthopaedics and Traumatology, Usak University, Usak, Turkey; 2 Orthopaedics and Traumatology Clinic, Esme State Hospital, Usak, Turkey

**Keywords:** Rotator cuff tear arthropathy, Shoulder prostheses, Shoulder replacement arthroplasties, Sleep quality, Kinesiophobia, Reverse shoulder arthroplasty

## Abstract

**Background:**

The current study aimed to determine the changes in pre-and post-operative Pittsburg sleep quality index (PSQI) and Tampa scale of kinesiophobia (TSK) values ​​according to the Hamada classification in patients who underwent reverse shoulder arthroplasty (RSA) for rotator cuff tear arthropathy (RCTA).

**Methods:**

One hundred and eight patients who underwent RSA for RCTA were reviewed retrospectively. The patients were divided into two groups with low grade (stages 1-2-3) (*n* = 49) and high grade (stages 4a-4b-5) (*n* = 59) according to the Hamada classification, which is the radiographic evaluation of RCTA. PSQI and TSK values ​​were calculated preoperatively, and post-operatively at the 6th week, 6th month, and 1st year. The change in PSQI and TSK values ​​between the evaluations and the effect of staging according to the Hamada classification on this change was examined.

**Results:**

When compared in preoperative evaluations, PSQI and TSK scores were found to be lower in low-grade group 1 (7.39 ± 1.56, 51.88 ± 4.62, respectively) than in high-grade group 2 (10.47 ± 2.39, 57.05 ± 3.25, respectively) according to Hamada classification (both *p* < 0.001). In the postoperative evaluations, PSQI and TSK results decreased gradually compared to the preoperative evaluations, and there was a severe decrease in both parameters between the 6th-week and 6th-month evaluations (both *p* < 0.001). Preoperatively, 102 (95%) patients had sleep disturbance (PSQI ≥ 6), and 108 (100%) patients had high kinesiophobia (TSK > 37). In the 1st year follow-ups, sleep disturbance was observed in 5 (5%) patients and kinesiophobia in 1 (1%) patient. When the Hamada stages were compared, it was seen that there was a significant difference before the operation (both *p* < 0.001), but the statistically significant difference disappeared in the PSQI value in the 1st year (*p* = 0.092) and in the TSK value in the 6th month (*p* = 0.164) post-operatively. It was observed that Hamada staging caused significant differences in PSQI and TSK values ​​in the preoperative period but did not affect the clinical results after treatment.

**Conclusions:**

RSA performed based on RCTA improves sleep quality and reduces kinesiophobia. RCTA stage negatively affects PSQI and TSK before the operation but does not show any effect after the treatment.

## Introduction

Neer et al. defined “cuff tear arthropathy” as humeral head collapse and glenohumeral joint disorganization due to massive rotator cuff tears [1, 2]. Reverse shoulder arthroplasty (RSA), first described by Beddow and Alloy in 1970, is now becoming increasingly common in the treatment of cuff arthropathy and traumatic injuries [3].

Many classification systems have been described in RCTA [4–7]. The Hamada system was used in the current study for the roentgenographic classification of cuff arthropathy patients [4]. According to this classification, acromio-humeral interval (AHI) > 6 mm in grade 1, <5 mm in grade 2, AHI < 5 mm with acetabularization of the acromion in grade 3, glenohumeral arthritis without acetabularization in grade 4A, glenohumeral arthritis with acetabularization in grade 4b, humeral head collapse in grade 5. In grades 4A, 4B, and 5, glenohumeral arthritis accompanies.

The current study evaluated the Pittsburg sleep quality index (PSQI) [8] and the Tampa Scale of Kinesiophobia (TSK) [9] in RSA patients. The PSQI was defined by Buysse et al. in 1988, it is used to evaluate sleep quality and disturbances over a one-month period. The total value of this measure, which consists of seven components and 19-item forms the PSQI score. An increase in the PSQI score indicates an increase in sleep disturbance (range of 0–21). A PSQI score of 6 and above have been reported to have 89.6% sensitivity and 85.6% specificity in distinguishing between good and poor sleep quality [8]. Kinesiophobia is defined as excessive avoidance of physical movement and activity due to fear of experiencing it again because of painful situations. Kori et al. 1990 defined the TSK as a measure that assesses fear of movement [9]. TSK is a 17-item method that measures fear of movement and re-injury. An increase in the TSK score indicates an increase in kinesiophobia (range of 17–68) [9, 10]. A TSK score of 37 and above has been reported to be associated with high kinesiophobia [11].

Weinberg et al. evaluated PSQI in their study without distinction of RCTA [12]. Many studies have been conducted on the relationship between shoulder pathologies, especially rotator cuff tears, and sleep quality [13–29]. However, no PSQI studies were found for RSA. Similarly, although there are studies of association with TSK in patients with rotator cuff tears [30, 31], no studies were found to indicate an association with RSA.

The current study aimed to determine the changes in pre-and post-operative PSQI and TSK values ​​according to the Hamada classification in patients who underwent RSA for RCTA.

## Materials and methods

### Selection of patients, ınclusion, and exclusion criteria

**Fig. 1:**
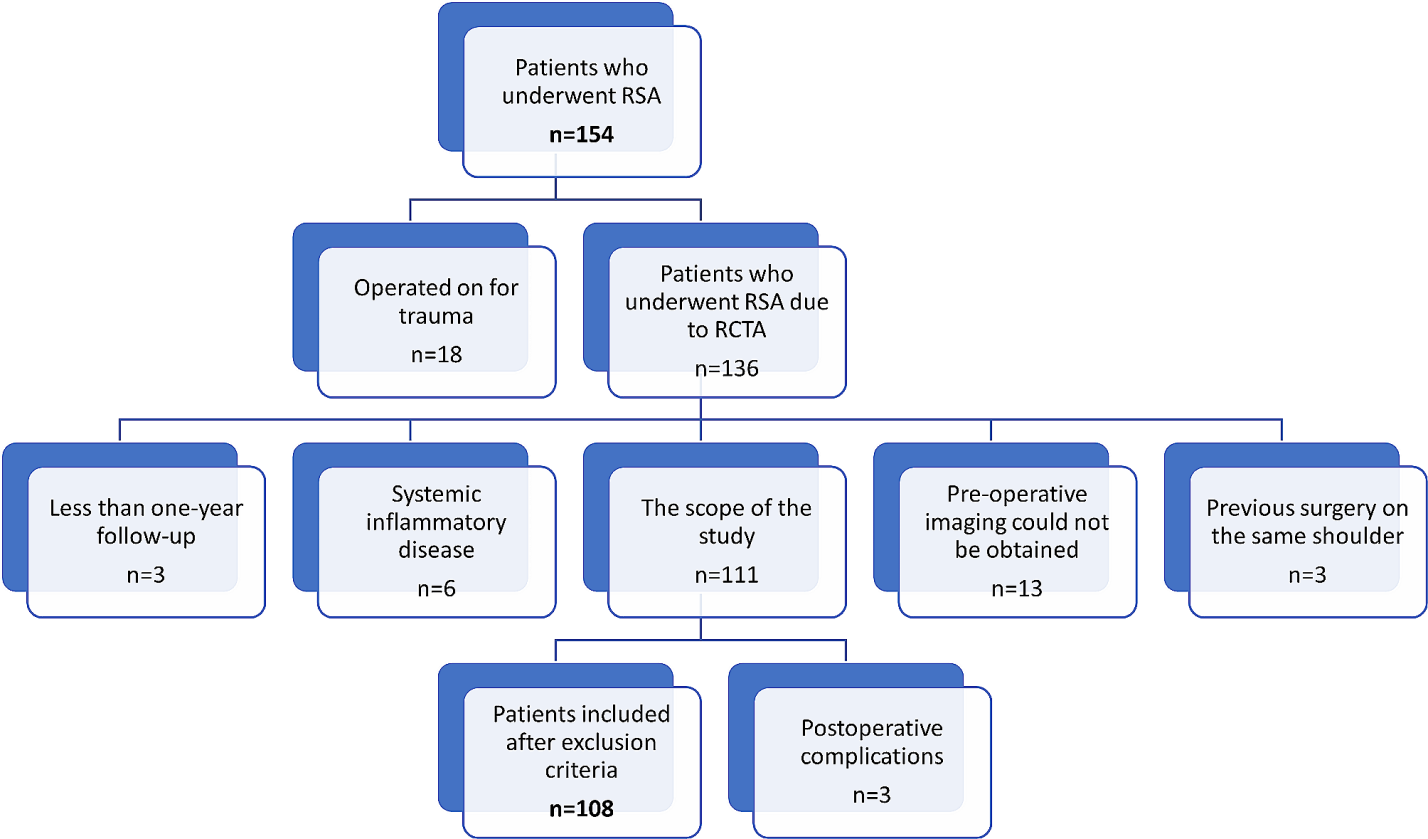
Flow chart of cases

Between 2015 and 2022, 154 patients who underwent RSA by the principal investigator were scanned retrospectively. Patients who had undergone RSA operation by the senior author in the last 8 years were determined as inclusion criteria. Patients whose pre-operative imaging could not be obtained (*n* = 13), who were operated on for trauma (*n* = 18), patients with postoperative complications (*n* = 3), with a previous history of surgery on the same shoulder (*n* = 3), less than one year follow-up (*n* = 3) and presence of systemic inflammatory disease (*n* = 6) were excluded from the study (Fig. 1). It was observed that some of the patients who operated due to trauma had pre-existing cuff arthropathy, while some did not. These patients were included in the exclusion criteria as they would be outside the main purpose of the study.

### Evaluated parameters

Pre-operative, post-operative 6th-week, 6th-month, and 12th-month PSQI and TSK values, pre-operative shoulder radiographs, and demographic data were obtained from the archive. Pre-operative shoulder radiographs of 108 (70%) patients included in the study were evaluated by two researchers on the same Picture Archiving and Communication Systems (PACS) independently according to the Hamada classification. It was evaluated whether PSQI and TSK values ​​differed in the pre-and post-operative evaluations and whether staging according to the Hamada classification affected these results. In addition, according to the Hamada classification, stages 1 (*n* = 9), 2 (*n* = 14), and 3 (*n* = 26) without glenohumeral arthritis (*n* = 49), and stages 4a (*n* = 16), 4b (*n* = 19), and 5 (*n* = 24) with accompanying glenohumeral arthritis (*n* = 59) were compared as separate groups. The images of the grade three case of Hamada, the most common type detected in the study, are given in Fig. 2 (a-b-c).

**Fig. 2:**
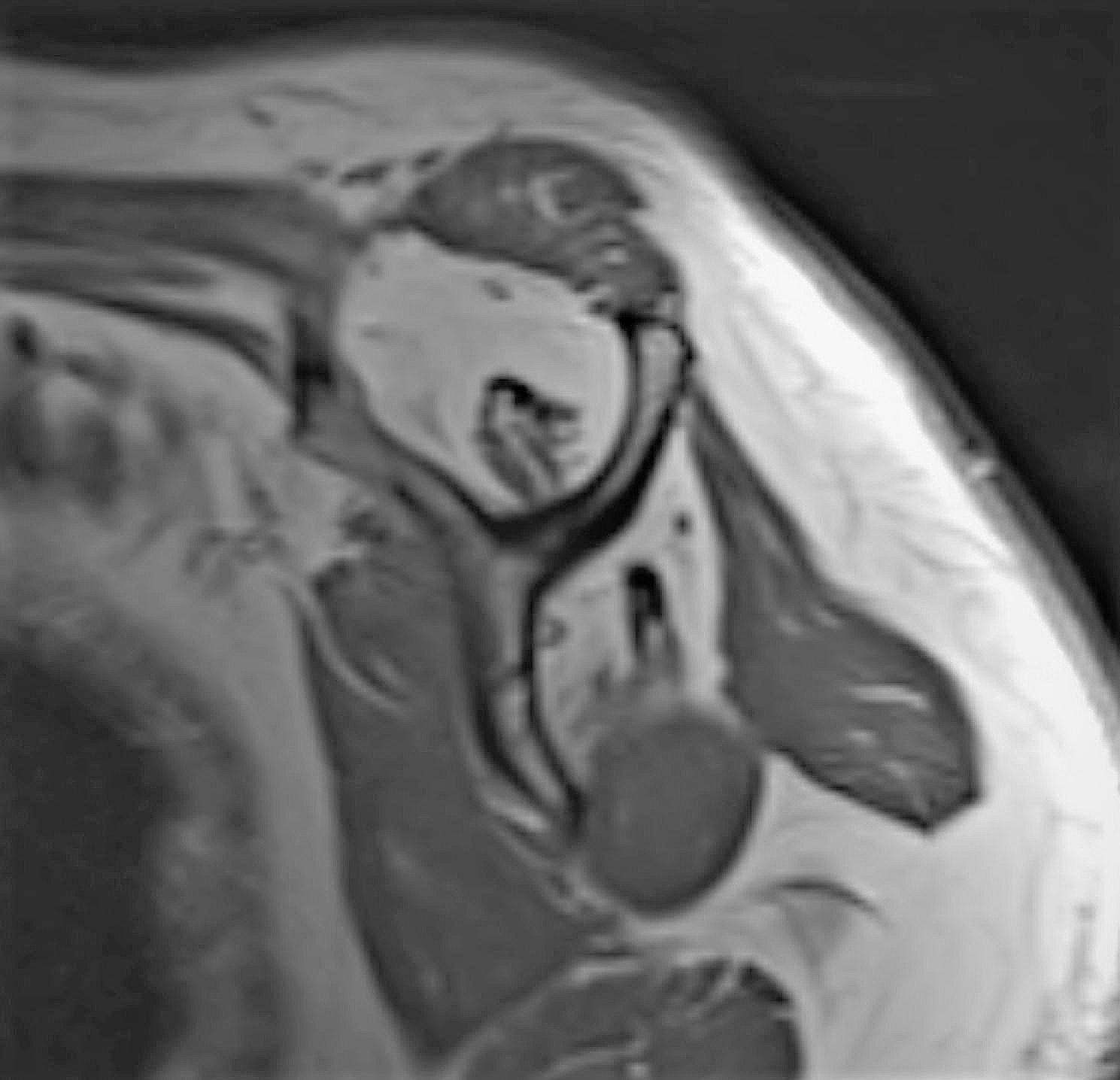
Images of a case sample of Hamada grade 3, the most common type detected in the study. 2a: T1-weighted oblique sagittal MRI section

**Fig. 2b:**
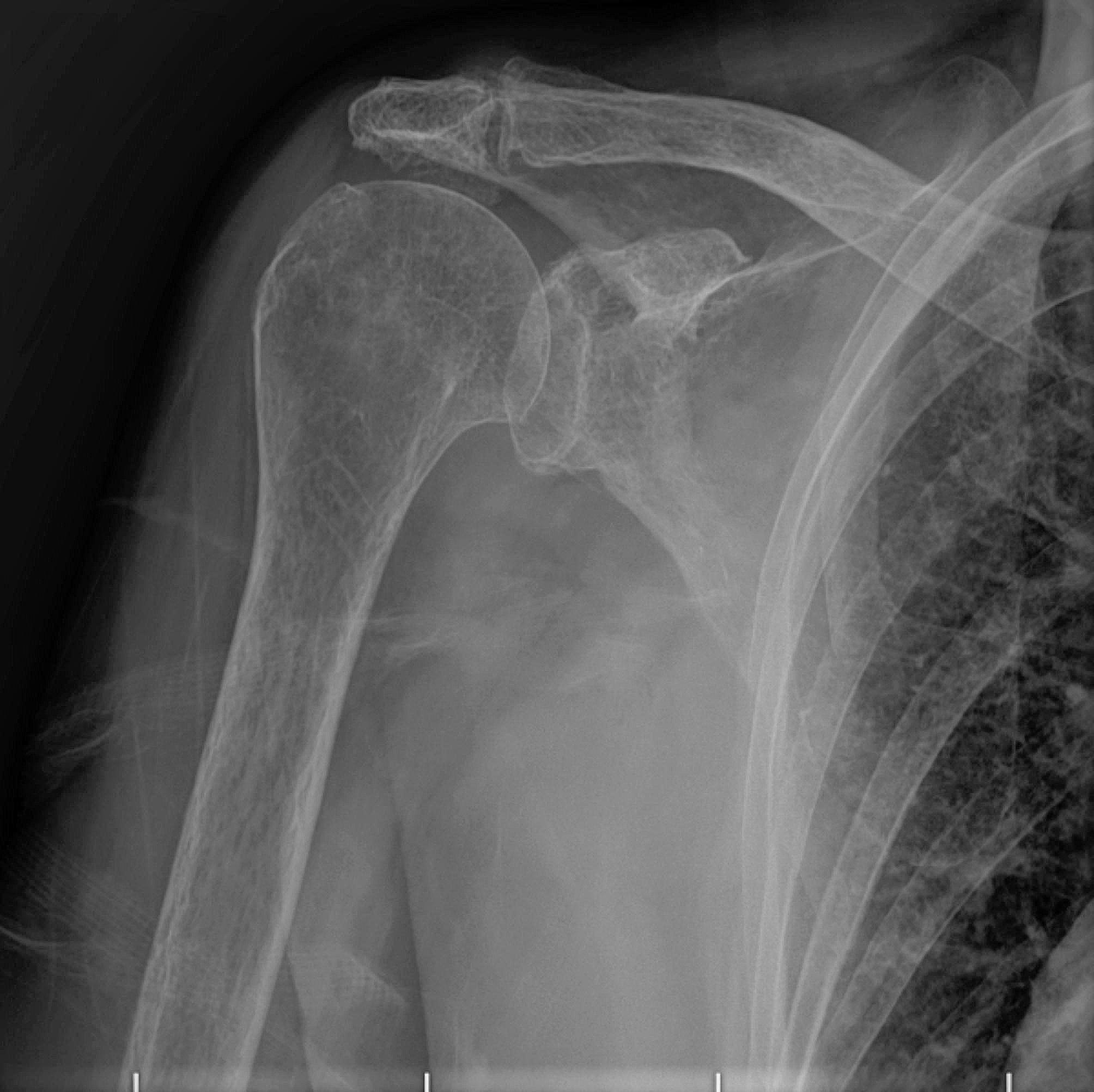
Pre-operative X-ray

**Fig. 2c:**
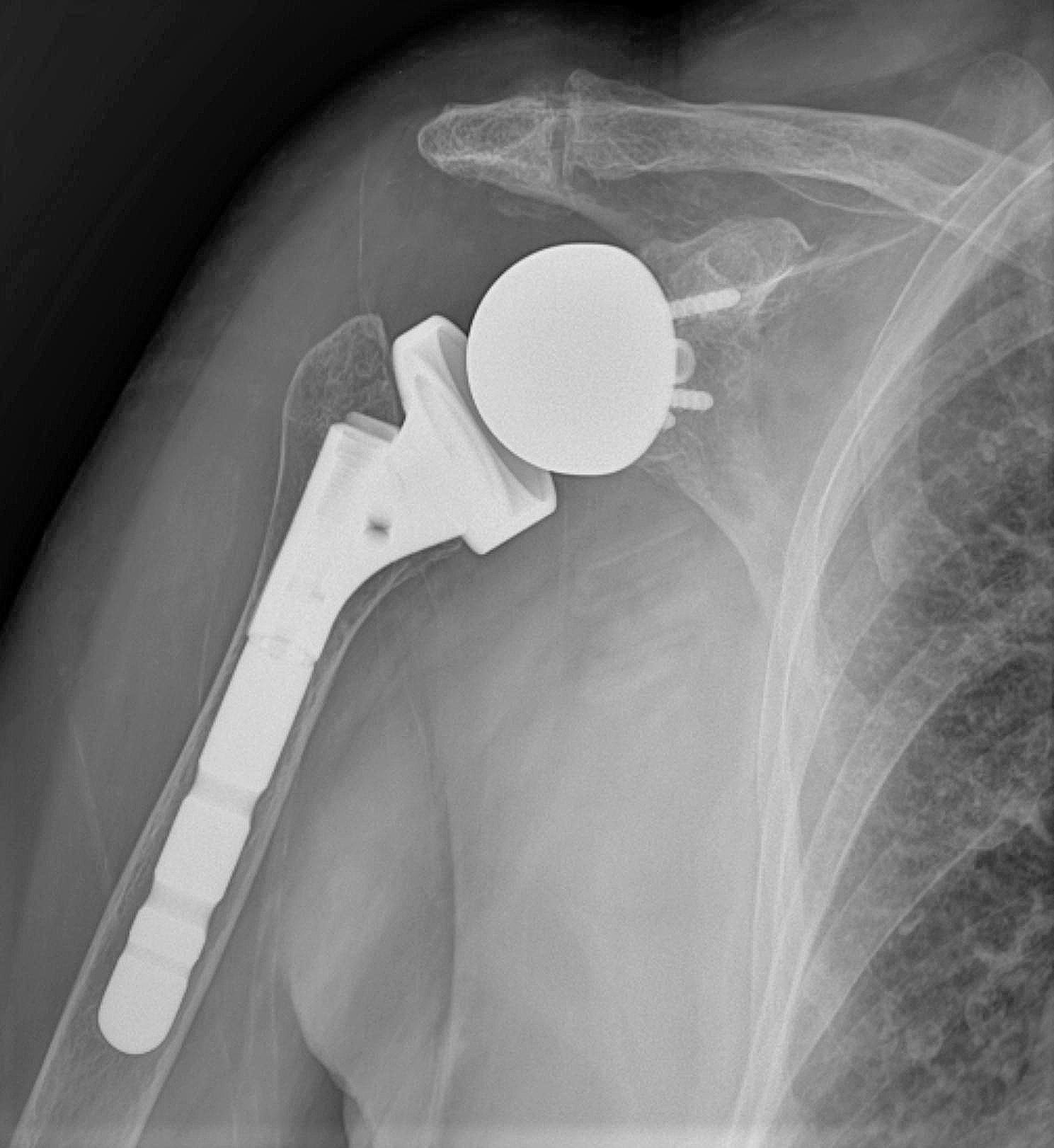
1st-year post-operative x-ray

### Statistical analysis

SPSS (Statistical Package for the Social Sciences) version 24 (IBM Corp., Armonk, New York, USA) was used for statistical analysis. Fisher’s Exact Test was used to compare categorical data. Shapiro Wilk test was applied to the measurements to be evaluated for normality analysis. Mann Whitney U analysis and Spearman Coefficient were used for non-normally distributed parameters, and an independent t-test and Pearson Coefficient were used for those with normal distribution. A p-value less than 0.05 was considered significant, an r-value below 0.3 was considered very weak, between 0.3 and 0.5 weak, 0.5–0.7 moderate, and above 0.7 strong correlation. Fleiss kappa (κ) coefficient was used for the inter-observer reliability. A κ value is always between 0 and 1; the higher k value indicates a better correlation. The κ values were graded as slight (0–0.2), fair (0.21–0.40), moderate (0.41–0.60), substantial (0.61–0.80), and almost perfect (0.81–1). Post hoc analysis was performed using the G*power 3.1.9.7 program (Heinrich-Heine-Universität Düsseldorf, GERMANY).

## Results

### Demographic data of the patients

The mean age of the 108 patients included in the study was 68.03 ± 5.11 (57–77). When they were divided into 2 groups according to the Hamada classification, the mean age of the 1st group, which included the 1st, 2nd, and 3rd stages, was 68.96 ± 4.86 (58–77), the mean age of the 2nd group, which included stages 4a, 4b and 5, was 67.25 ± 5.23 (57–76) years (*p* = 0.091). Seventy-nine (73%) of the patients included in the study were female. According to the Hamada classification, there were 32 (65%) women in the 1st group and 47 (80%) women in the 2nd group (*p* = 0.127). It was seen that 84 (78%) of the patients had been operated on in their dominant extremity. According to the groups, 36 (73%) patients in group 1 and 48 (81%) patients in group 2 were found to have disease in the dominant extremity (*p* = 0.360). The mean body mass index (BMI) was 27.32 ± 2.24 (22-32). According to the Hamada groups, the mean BMI of the 1st group was 27.65 ± 2.35 (22-32), while the mean of the 2nd group was 27.05 ± 2.67 (23-32) (*p* = 0.124) (Table 1).

**Table 1:** Demographic data of the patients

	Without Glenohumeral Arthritis	Accompanying Glenohumeral Arthritis	p-value	Total
Age mean ± SD (min-max)	68.96 ± 4.86 (58-77)	67.25 ± 5.23 (57-76)	0.091	68.03 ± 5.11 (57-77)
Sex male/female (n)	17/32	12/47	0.127	29/79
Dominant/non-dominant arm (n)	36/13	48/11	0.360	84/24
BMI mean ± SD (min-max)	27.65 ± 2.35 (22–32)	27.05 ± 2.67 (23–32)	0.124	27.32 ± 2.24 (22–32)
n	49	59		108

SD: Standard deviation, BMI: body mass index

### Evaluation of PSQI values

The mean pre-operative PSQI score was 9.07 ± 2.56 (4-16). When evaluated on a group basis according to the Hamada classification, the mean pre-operative PSQI score in the 1st group (stages 1, 2, 3) was 7.39 ± 1.56, while it was 10.47 ± 2.39 in the 2nd group (stages 4a, 4b, 5) (*p* < 0.001). It was seen that post-operative mean PSQI scores were 8.49 ± 2.56 (3-16) in the 6th week (1st group 7.02 ± 1.51, 2nd group 9.71 ± 2.61, *p* < 0.001), 4.33 ± 1.32 (2-8) in the 6th month (3.98 ± 0.97, 4.63 ± 1.50, respectively, *p* = 0.032), and 4.14 ± 1.17 (1-7) at 1-year follow up (3.90 ± 0.92, 4.34 ± 1.32, respectively, *p* = 0.092) (Table 2).

**Table 2:** Intergroup comparison of PSQI assessments

	Without Glenohumeral Arthritis mean ± SD	Accompanying Glenohumeral Arthritis mean ± SD	p-value	Total mean ± SD (min-max)
1st eval.	7.39 ± 1.56	10.47 ± 2.39	**< 0.001**	9.07 ± 2.56 (4–16)
2nd eval.	7.02 ± 1.51	9.71 ± 2.61	**< 0.001**	8.49 ± 2.56 (3–16)
3rd eval.	3.98 ± 0.97	4.63 ± 1.50	**0.032**	4.33 ± 1.32 (2–8)
4th eval.	3.90 ± 0.92	4.34 ± 1.32	0.092	4.14 ± 1.17 (1–7)

PSQI: Pittsburg sleep quality index, 1st : pre-operatively, 2nd : post-operative 6th week, 3rd : post-operative 6th month, 4th : post-operative 1-year, eval: evaluation, SD: standard deviation

Buyyse et al. [8] according to the data they defined (PSQI ≥ 6 is sleep disturbance), sleep disturbance was detected in 102 (95%) patients before the operation, it was found to have decreased to 98 (91%) patients at the 6th-week, 16 (15%) patients at the 6th-month, and to five (5%) patients at the 1st-year follow-up. In the inter-evaluation examination, there was no statistical difference between pre-operative and 6 weeks (*p* = 0.074), a high level of significant difference was observed in the comparison of 6 weeks and 6 months (*p* < 0.001), and the difference disappeared again between 6 months and 1 year (*p* = 0.321) (Table 3).

**Table 3:** Comparisons of PSQI assessments within the group and on all patients

p-value			
	**Without Glenohumeral Arthritis**	**Accompanying Glenohumeral Arthritis**	**Total**
1st – 2nd eval.	0.240	0.146	0.074
2nd – 3rd eval.	**< 0.001**	**< 0.001**	**< 0.001**
3rd – 4th eval.	0.660	0.315	0.321

PSQI: Pittsburg sleep quality index, 1st : pre-operatively, 2nd : post-operative 6th week, 3rd : post-operative 6th month, 4th : post-operative 1-year, eval: evaluation

Considering the correlation between the stage and PSQI score according to the Hamada classification (stages 1, 2, 3, 4a, 4b, 5), it was observed that the high correlation between the preoperative stage and the score decreased in the follow-ups (*r* = 0.708, *p* < 0.001 pre-operatively [strong correlation], *r* = 0.630, *p* < 0.001 postoperative 6th week [moderate correlation], *r* = 0.259, *p* = 0.007 postoperative 6th month [very weak correlation], *r* = 0.243, *p* = 0.011 postoperative 1st year [very weak correlation], respectively) (Table 4).

**Table 4:** Correlation of PSQI and TSK values with Hamada stages based on evaluations

	PSQI		TSK	
	r-value	interpret.	r-value	interpret.
1st eval.	0.708	strong corr.	0.680	moderate corr.
2nd eval.	0.630	moderate corr.	0.480	weak corr.
3rd eval.	0.259	very weak corr.	0.060	very weak corr.
4th eval.	0.243	very weak corr.	− 0.335	weak corr.

PSQI: Pittsburg sleep quality index, TSK: Tampa Scale of Kinesiophobia, 1st : pre-operatively, 2nd : post-operative 6th week, 3rd : post-operative 6th month, 4th : post-operative 1-year, eval: evaluation, interpret: interpretation, corr: correlation

It was determined that the increase in BMI and the increase in the preoperative PSQI score were moderately correlated (*r* = 0.646, *p* < 0.001).

### Evaluation of TSK values

The mean pre-operative TSK score was 54.70 ± 4.69 (38–64). In terms of groups, it was found that the mean in the 1st group (stages 1, 2, 3) was 51.88 ± 4.62, while it was 57.05 ± 3.25 in the second group (stages 4a, 4b, 5) (*p* < 0.001). It was seen that post-operative mean TSK scores were 53.24 ± 5.36 (20–61) in the 6th week (51.14 ± 4.64, 54.98 ± 5.34, groups respectively, *p* < 0.001), 30.13 ± 4.18 (23–41) at the 6th month (29.57 ± 4.32, 30.59 ± 4.04, respectively, *p* = 0.164), and 27.83 ± 2.33 (27–41) at 1-year follow up (28.33 ± 2.82, 27.42 ± 1.76, respectively, *p* = 0.060) (Table 5).

**Table 5:** Intergroup comparison of TSK assessments

	Without Glenohumeral Arthritis mean ± SD	Accompanying Glenohumeral Arthritis mean ± SD	p-value	Total mean ± SD (min-max)
1st eval.	51.88 ± 4.62	57.05 ± 3.25	**< 0.001**	54.70 ± 4.69 (38–64)
2nd eval.	51.14 ± 4.64	54.98 ± 5.34	**< 0.001**	53.24 ± 5.36 (20–61)
3rd eval.	29.57 ± 4.32	30.59 ± 4.04	0.164	30.13 ± 4.18 (23–41)
4th eval.	28.33 ± 2.82	27.42 ± 1.76	0.060	27.83 ± 2.33 (27–41)

TSK: Tampa Scale of Kinesiophobia, 1st : pre-operatively, 2nd : post-operative 6th week, 3rd : post-operative 6th month, 4th : post-operative 1-year, eval: evaluation, SD: standard deviation

It was observed that 108 (100%) patients had high kinesiophobia (TSK > 37) [11] before the operation and at the 6th-week controls, and it decreased to six (6%) patients with a serious decrease in the 6th month. In the 1st year follow-up, it was learned that one patient (1%) experienced high kinesiophobia, and this patient had a score of 33 in the 6th-week evaluation but had trauma during this period. No statistically significant difference existed between the pre-operative and 6th-week controls in the TSK score in the low-stage group (stages 1, 2, 3) (*p* = 0.219). In contrast, this difference was found to be statistically significantly decreased in the high-stage group (stages 4a, 4b, 5) (*p* = 0.012). In the 6th-week and 6th-month comparisons, it decreased significantly in both groups (both *p* < 0.001), in the 6th-month and 1st-year comparisons, it decreased significantly in the high-stage group (*p* < 0.001), and the decrease in the low-grade group was not statistically significant (*p* = 0.313) (Table 6).

**Table 6:** Comparisons of TSK assessments within the group and on all patients

p-value			
	**Without Glenohumeral Arthritis**	**Accompanying Glenohumeral Arthritis**	**Total**
1st – 2nd eval.	0.219	**0.012**	0.062
2nd – 3rd eval.	**< 0.001**	**< 0.001**	**< 0.001**
3rd – 4th eval.	0.313	**< 0.001**	**< 0.001**

TSK: Tampa Scale of Kinesiophobia, 1st : pre-operatively, 2nd : post-operative 6th week, 3rd : post-operative 6th month, 4th : post-operative 1-year, eval: evaluation

When the correlation of Hamada stages with TSK score was examined, moderate correlation in preoperative evaluation (*r* = 680, *p* < 0.001), weak at 6th-weeks (*r* = 0.480, *p* < 0.001, [weak correlation]), and it was observed that the 6th-month was very weak (*r* = 0.060, *p* = 0.540). In contrast, in the 1st-year evaluation, a negative weak correlation (*r*=-0.335, *p* < 0.001) was detected (Table 4).

A very weak correlation was found between BMI and preoperative TSK score (*r* = 0.076, *p* = 0.436).

### Reliability and post hoc analysis

Moderate inter-observer agreement was determined in the interpretation of Hamada stages on X-rays (k = 0.518).

Power (1-*b*) in post hoc analysis was calculated as 0.806 (sample size [n1 = 49 – n2 = 59], effect size d = 0.5, and *a* error probability = 0.05).

## Discussion

Pain is a symptom that seriously affects the quality of sleep and life [32, 33]. Shoulder pain lasting more than 3 months is a strong indicator of sleep disturbance, close relationships between depression-anxiety and sleep disorders have been reported [33]. In the current study, we aimed to determine the changes in PSQI and TSK values ​​according to the Hamada classification in patients who underwent RSA due to RCTA. When both groups were compared in preoperative controls, PSQI and TSK scores were found to be lower in the low-grade 1st group (stages 1-2-3) (without glenohumeral arthritis) compared to the high-grade 2nd group (stages 4a-4b-5) (accompanying glenohumeral arthritis) (both *p* < 0.001). PSQI and TSK results were observed to decrease gradually in the post-operative evaluations compared to the preoperative ones. In PSQI analysis, in the comparative evaluation of both groups, a statistically significant difference was observed in the 6th-week and 6th-month comparisons (*p* < 0.001, 0.032, respectively) as in the preoperative control (*p* < 0.001), while in the 1st year comparison, it was observed that the statistical difference disappeared (*p* = 0.092). When the TSK was examined, it was observed that the statistically significant difference in the evaluations between the groups continued in the 6th-week control as well as in the pre-operative control (both *p* < 0.001), while the statistically significant difference disappeared in the 6th month and 1st-year controls (*p* = 0.164, 0.060, respectively). In addition, a severe decrease was detected between the 6th-week and 6th-month evaluations of both PSQI and TSK scores (both *p* < 0.001).

Weinberg et al. [12] evaluated PSQI in 42 anatomic and 32 reverse total shoulder arthroplasty (TSA) operation patients. They reported that 84% of the patients had preoperative sleep disturbance and the mean PSQI score was 10.1. In addition, they reported that there was no correlation between TSA type (reverse or anatomic) and PSQI score, while the PSQI score gradually decreased in the post-operative follow-ups, regardless of the subgroups (6-weeks = 7.7, 3-months = 6.1, 6-months = 5.7, 1-year = 4.3). In the current study, in which we evaluated only RSA patients after RCTA, the mean pre-operative PSQI value was 9.07 ± 2.56 and 95% of the patients had sleep disturbances. In addition, it was observed that the PSQI value decreased gradually in the postoperative follow-ups and was 4.14 ± 1.17 in the 1st-year controls. The results we found in a series of 108 patients are consistent with this study.

Morris et al. [16] in a study evaluating sleep disturbance with anatomical TSA, it was reported that 91% of the patients had sleep disturbance before the operation, and this rate decreased to 20% after the operation. Similarly, Austin et al. [28] reported the mean PSQI value as 11.70 in rotator cuff lesions. In the same study, they showed that 89% of rotator cuff tear patients had sleep disturbance. They found that with arthroscopic rotator cuff repair, the PSQI score was statistically significantly reduced compared to the control group. In the current study, it was observed that the rate of sleep disturbance, which was 95% pre-operatively, decreased to 15% in the 6th month and to 5% in the 1st year. A significant decrease was observed especially in the comparison of 6 weeks and 6 months (*p* < 0.001).

Kolade et al. [35] in their study on TSA patients, found that the sleep score showed a gradual improvement in the postoperative period compared to the preoperative score and showed an improvement of 104% in the 3rd-month control. In the present study, although there was a slight decrease between the pre-operative and 6-week evaluation (*p* = 0.074), it was observed that sleep quality increased by 109% in the 6th month and 119% in the 1st year compared to the pre-operative value.

Wang et al. [30] in their study in which they examined kinesiophobia in 49 patients with rotator cuff tears, reported that the score decreased in long-term follow-ups. In the current study evaluating RSA patients, it was observed that the TSK score decreased by 45% in the 6th month and by 49% in the 1st year. When all patients were evaluated, it was observed that the TSK decreased statistically between the 6th week and the 6th month and between the 6th month and the 1st year (both *p* < 0.001).

Kääb et al. [36] in their study in which they evaluated RSA in massive rotator cuff ruptures, stated that the stage did not affect clinical outcomes or complications according to the Hamada classification. In the current study, patients who developed complications were among the exclusion criteria, and sleep quality and kinesiophobia were evaluated as clinical outcomes. When compared according to the stage of Hamada, it was observed that PSQI and TSK scores in the preoperative period showed a high correlation with the stage (*r* = 0.708, 0.680, respectively), and this rate decreased in the 1st year follow-up (*r* = 0.243, 0.-335, respectively). When the stages were compared as a group (1-2-3, 4a-4b-5), it was seen that there was a significant difference before the operation (both *p* < 0.001), but the statistically significant difference disappeared in the PSQI value in the 1st year (*p* = 0.092) and in the TSK value in the 6th month (*p* = 0.164) post-operatively. These results showed that Hamada staging caused significant differences in PSQI and TSK values ​​in the pre-operative period but did not affect the clinical outcomes after treatment. Kappe et al. [37] showed moderate inter-observer reliability for the Hamada classification (k:0.407), similarly, in this study, a moderate agreement was found between observers (k = 0.518).

### Limitation

This study has some limitations. The main limitation of the study is its retrospective nature. Another limitation is the use of only radiographic Hamada staging when performing RCTA classification. It would have been better to include the 3rd-month evaluation in addition to the 6th-week, 6th-month, and 1st-year, but it was not possible due to retrospective planning. Since the duration of the patient’s preoperative complaints was unknown, it could not be included. Another limitation is that there was no evaluation of the upper extremity functional level. The fact that psychological evaluation, which is important among the problems affecting sleep quality, was not performed before the operation was also determined as a limitation.

## Conclusion

This study found that RSA performed due to RCTA improved sleep quality and reduced kinesiophobia. As the Hamada stage progresses, the increasing level of glenohumeral arthritis increases kinesiophobia and disrupts sleep quality, in addition, due to low mobility, it may cause relatively more difficulty during surgery. However, after RSA treatment, it was determined that the level of glenohumeral arthritis before the operation did not make a difference in the results, it was observed that kinesiophobia decreased and sleep quality improved in all patients. While PSQI and TSK and Hamada stages were highly correlated before the operation, the correlation decreased after the treatment.

## Data Availability

The corresponding author can provide data when necessary.

## Abbreviations

PSQI: Pittsburg sleep quality index
TSK: Tampa scale of kinesiophobia
RSA: Reverse shoulder arthroplasty
RCTA: Rotator cuff tear arthropathy
AHI: Acromio-humeral interval
